# Scaly MoS_2_/rGO Composite as an Anode Material for High-Performance Potassium-Ion Battery

**DOI:** 10.3390/molecules29132977

**Published:** 2024-06-22

**Authors:** Bin Wang, Tao Deng, Jingjing Liu, Beibei Sun, Yun Su, Ruixia Ti, Lihua Shangguan, Chaoyang Zhang, Yu Tang, Na Cheng, Yan Xu, Junling Guo

**Affiliations:** 1 School of Physics and Electronic Engineering, Xinxiang University, Xinxiang 453003, China; wangbin2013@xxu.edu.cn (B.W.); dengtaoxuyiyin@163.com (T.D.); bit-1@163.com (Y.S.); tiruixia@yeah.net (R.T.);; 2 School of Mechanical Engineering, Chengdu University, Chengdu 610106, China; 3 Henan Province Engineering Research Center of New Energy Storage System, Xinxiang University, Xinxiang 453003, China; 4 Country State Center for International Cooperation on Designer Low-Carbon & Environmental Materials, School of Materials Science and Engineering, Zhengzhou University, 100 Kexue Avenue, Zhengzhou 450001, China

**Keywords:** MoS_2_/rGO, potassium-ion battery, anode material, scaled structure

## Abstract

Potassium-ion batteries (PIBs) have been widely studied owing to the abundant reserves, widespread distribution, and easy extraction of potassium (K) resources. Molybdenum disulfide (MoS_2_) has received a great deal of attention as a key anode material for PIBs owing to its two-dimensional diffusion channels for K^+^ ions. However, due to its poor electronic conductivity and the huge influence of embedded K^+^ ions (with a large ionic radius of 3.6 Å) on MoS_2_ layer, MoS_2_ anodes exhibit a poor rate performance and easily collapsed structure. To address these issues, the common strategies are enlarging the interlayer spacing to reduce the mechanical strain and increasing the electronic conductivity by adding conductive agents. However, simultaneous implementation of the above strategies by simple methods is currently still a challenge. Herein, MoS_2_ anodes on reduced graphene oxide (MoS_2_/rGO) composite were prepared using one-step hydrothermal methods. Owing to the presence of rGO in the synthesis process, MoS_2_ possesses a unique scaled structure with large layer spacing, and the intrinsic conductivity of MoS_2_ is proved. As a result, MoS_2_/rGO composite anodes exhibit a larger rate performance and better cycle stability than that of anodes based on pure MoS_2_, and the direct mixtures of MoS_2_ and graphene oxide (MoS_2_-GO). This work suggests that the composite material of MoS_2_/rGO has infinite possibilities as a high-quality anode material for PIBs.

## 1. Introduction

Energy remains a crucial aspect in the current state of global development. Developing sustainable and clean energy is an inevitable trend, particularly given the massive consumption of traditional fossil fuels and the deteriorating global environment. Electrochemical energy storage (EES) systems are becoming increasingly popular in the field of energy technology [1,2,3]. Lithium-ion batteries (LIBs) are extensively deployed in a wide range of consumer electronics, electric vehicles, and national grids due to their high energy density and long cycle life in various EES systems. However, lithium is not an abundant resource in the Earth’s reserves, accounting for only 0.0017 wt%. Furthermore, its uneven distribution leads to high extraction and transportation costs, making it a poorly suited sustainable option for meeting the world’s long-term energy needs [4,5].

In recent years, research on batteries using Na, Mg, Al, and K has made considerable progress and received increasing attention [6,7,8,9]. Among them, potassium-ion batteries (PIBs) have been widely studied owing to the abundant K element in nature (2.09 wt%), high energy density (200–300 Wh kg^−1^), and favorable K^+^ ions mobility in nonaqueous electrolytes (resulting from the smaller Stokes radius of K^+^ compared to Li^+^ or Na^+^) [10,11,12]. However, due to the inherent large ionic radius of K^+^ ion (3.6 Å), it encounters high diffusion barriers, and easily cause the material structure to collapse during insertion/extraction. Therefore, it is crucial to find electrode materials that can allow speed kinetics and maintain structural stability during K^+^ de-intercalation [13,14,15].

Noncarbon-based intercalation materials, in particular molybdenum disulfide (MoS_2_), as typical transition metal disulfides (TMDs), have received a great deal of attention as a key anode material for PIBs [16,17]. MoS_2_ is an economical and readily available mineral with a layered structure, which enables it to open up two-dimensional diffusion channels during K^+^ intercalation [18,19,20]. However, the rate performances and electrochemical stability of MoS_2_ is still low due to its poor electronic conductivity (σ_RT_ = ~10^−4^ s cm^−1^) and the huge influence of embedded K^+^ ions on MoS_2_ layer [21]. Many research studies have been carried out to solve these problems. The common strategies are increasing the electrical conductivity by adding conductive agents and enlarging the interlayer spacing to reduce the mechanical strain [22,23,24]. Graphene oxide (GO) is an electrically conductive layered graphite oxide whose surface is rich in a variety of functional groups, which can provide an abundance of effective active sites [25]. However, an excess of functional groups can cause it to become too active to maintain a stable structure. Reduced graphene oxide (rGO), as a product of GO reduction, has higher electrical conductivity and structural stability while retaining functional groups. In addition, it was found that rGO can be used as a substrate material to modulate the microstructure of other materials [26]. However, simultaneous implementation of the above strategies by simple methods is currently still a challenge.

In this work, we obtained MoS_2_ on reduced graphene oxide (MoS_2_/rGO) composite by the one-step hydrothermal method. According to our experiments and calculations, MoS_2_ in such composite has an ultrathin scale-like structure with enlarged interlayer spacing, which indirectly increases the specific surface area, provides more active sites for K^+^, and ensures structural stability. In addition, the incorporation of rGO can increase the intrinsic conductivity of MoS_2_. As a result, MoS_2_/rGO composite anodes exhibited a better rate performance (287.15, 266.7, 220.3, and 184.6 mAh g^−1^, at 50, 100, 500, and 1000 mA g^−1^, respectively) and cycle stability (99% capacity retention after 100 cycles at 500 mA g^−1^) than that of anodes based on pure MoS_2_, and the direct mixtures of MoS_2_ and graphene oxide (MoS_2_-GO). This work demonstrates the possibility of using MoS_2_/rGO composite as anodes for PIBs.

## 2. Results and Discussion

**Figure 1:**
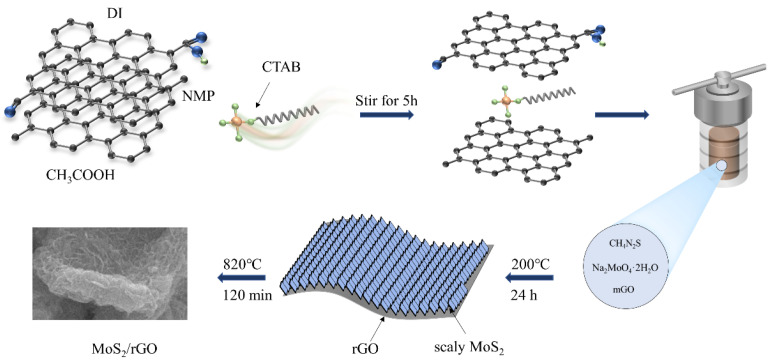
Schematic showing the synthesis of MoS_2_/rGO composite.

The preparation of scaly MoS_2_/rGO composite by a one-step reaction is illustrated in Figure 1. First, a typical modification method for GO is used [27]. The ammonium ions of cetyltrimethylammonium bromide (CTAB), which are positively charged, can attract the carboxyl group of GO, which has a negative charge, through Coulombic forces. The isolation of the lamellar GO sheets is a consequence of CTAB insertion. Next, a composite of MoS_2_/rGO is synthesized in a solvothermal reaction of Na_2_MoO_4_·2H_2_O and CH_4_N_2_S at 200 °C with the presence of rGO. There are several oxygen-containing groups in rGO, which are the adsorption sites for Mo^4+^ and K^+^, and also the nucleation sites for nanoparticles. As a result, uniform MoS_2_ precursors develop on the surface of rGO. The Mo-O-C bonds located at the remaining oxygen sites can interact with van der Waals forces present among the partial rGO, ultimately leading to the binding of the ions and partial rGO. The MoS_2_/rGO precursor shall undergo annealing treatment to yield MoS_2_/rGO composite.

**Figure 2:**
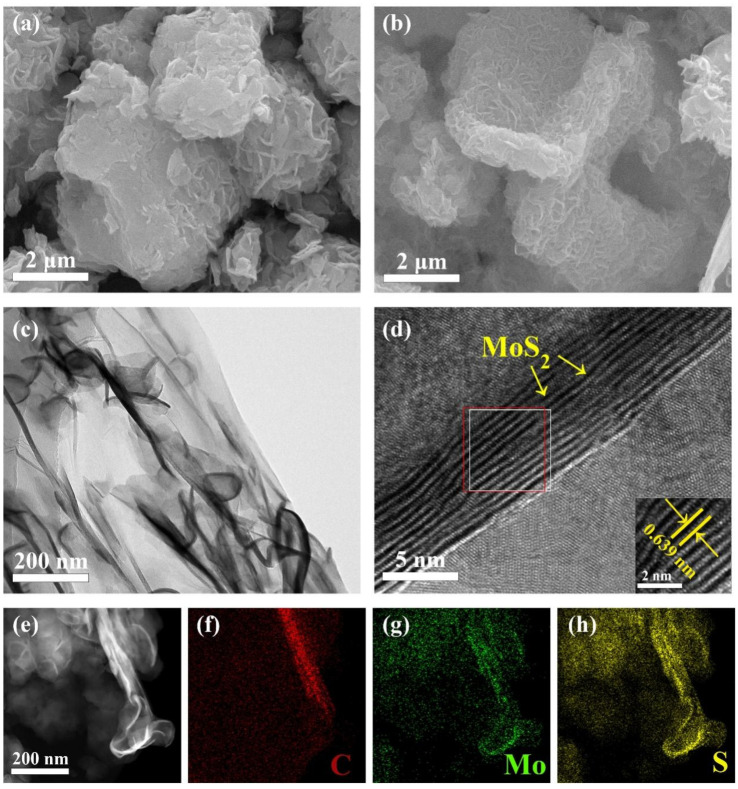
(**a**) SEM image of the MoS_2_, (**b**) SEM image of the MoS_2_/rGO composite, (**c**) TEM image of MoS_2_/rGO composite, and the image in the lower right-hand corner of the image is an enlargement of the red and white boxes, (**d**) HRTEM image of MoS_2_/rGO, (**e**) STEM image of MoS_2_/rGO composite, (**f**–**h**) the mapping map of the corresponding element of MoS_2_/rGO composite.

Scanning electron microscopy (SEM) was employed to examine the morphology of MoS_2_ and MoS_2_/rGO composite. As illustrated in Figure 2a,b, some of the MoS_2_ nanosheets exhibit a tendency to agglomerate. With regard to the MoS_2_/rGO composite, it can be observed that the scaly MoS_2_ nanosheets are uniformly distributed on the MoS_2_/rGO surface and are thinner and smaller in size. This result indicates that the presence of rGO effectively inhibits the agglomeration of MoS_2_ nanosheets. The scaly MoS_2_ nanosheets can enhance the specific surface area of MoS_2_/rGO and facilitate the generation of more active sites for K^+^ [28]. The microstructure of MoS_2_/rGO composite was investigated by field emission transmission electron microscopy (TEM). Figure 2c shows a magnified TEM image of MoS_2_/rGO at 200 nm scale, which clearly demonstrates MoS_2_ nanosheets embedded in translucent rGO sheets. Notably, the molybdenum disulfide nanosheets exhibit multilayered stacked lattice stripes, indicating a lattice spacing of 6.39 Å. This spacing is larger than the crystalline hexagonal 2H-MoS_2_ lattice spacing (002) of 6.1554 Å, as shown in Figure 2d [29]. To further support the observations in Figure 2c, Figure 2e shows scanning transmission electron microscopy (STEM) images of MoS_2_/rGO, providing additional evidence that the appearance of MoS_2_ scales is a result of MoS_2_ wrapped around rGO. Figure 2f–h show the total and partial elemental maps of MoS_2_/rGO captured at 200 nm scale. These maps reveal the uniform distribution of carbon (C), molybdenum (Mo), and sulfur (S) in the composite. This uniform distribution further confirms the successful preparation of MoS_2_/rGO composite and supports the conjecture that MoS_2_ grows on rGO.

**Figure 3:**
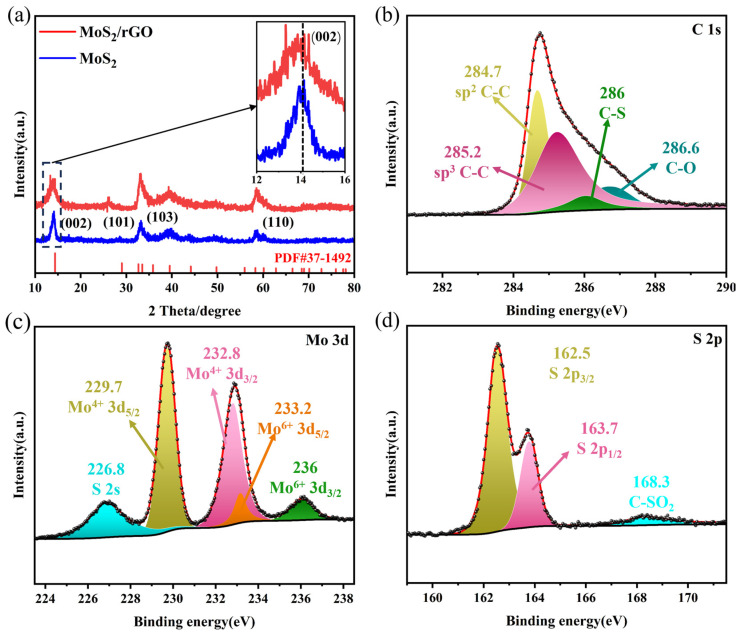
(**a**) XRD patterns of the MoS_2_/rGO composite and MoS_2_, (**b**) XPS spectra of C 1s, (**c**) XPS spectra of Mo 3d, (**d**) XPS spectra of S 2p.

The crystal structure of the synthesized MoS_2_/rGO sample was analyzed using X-ray powder diffraction (XRD) patterns. Figure 3a presents the XRD diffractograms of MoS_2_/rGO and MoS_2_. The four major diffraction peaks, observed at approximately 14.3°, 33.5°, 39.5°, and 58.3°, respectively, correspond to the (002), (101), (103), and (110) crystallographic planes of 2H-MoS_2_ (PDF#37-1492) [30]. The disappearance of the principal diffraction peak of GO at approximately 12° and the emergence of the principal diffraction peak of rGO at approximately 26° indicate that GO was successfully reduced to rGO [31]. Notably, the peak of MoS_2_/rGO at the (002) crystal plane is shifted to the left, indicating an enlarged layer spacing. The expanded layer spacing facilitates potassium insertion and extraction during the cycling process, thereby enhancing the potassium storage capacity of MoS_2_/rGO.

X-ray photoelectron spectroscopy (XPS) was utilized to determine the elemental composition and chemical bonding in MoS_2_/rGO. The spectrum of C 1s can be divided into four peaks, 284.7, 285.2, 268, and 286.6 (Figure 3b), which correspond to the sp2-banded of C-C, sp3-banded of C-C, C-S, and C-O, respectively [26]. As illustrated in Figure 3c, the peak at 226 in the Mo 3d spectrum is attributed to S 2s [32], while the pair of characteristic peaks at 229.7 and 232.8 are attributed to Mo 3d_5/2_ and Mo 3d_3/2_, respectively. This indicates that the valence state of Mo is +4 [33]. The pair of characteristic peaks at 232.2 and 236 should be attributed to Mo 3d_5/2_ and Mo 3d_3/2_ for Mo^6+^, which may be attributed to the oxidation of the MoS_2_ surface, resulting in the change to Mo^6+^ [34]. Figure 3d depicts the spectrum of S 2p. The pair of characteristic peaks at 162.5 and 163.7 eV can be attributed to the S 2p_3/2_ and S 2p_1/2_ of S^2−^. The small peak at 168.3 eV is indicative of the S-O-C bond [35]. A comparison of the XPS of MoS_2_ and MoS_2_-GO in Figure S1 reveals that pure MoS_2_ and MoS_2_-GO lack the C-S bond observed in the C 1s and S 2p spectra. This suggests that rGO and MoS_2_ can be more effectively bonded together in the MoS_2_/rGO material, leading to enhanced stability. Furthermore, the thermogravimetric analysis (TGA), as shown in Figure S2, was used to evaluate the MoS_2_ content of MoS_2_/rGO. The weight loss in the temperature range 300–600 °C is due to the decomposition of rGO and oxidation reaction of MoS_2_ to MoO_3_. Thus, the content of MoS_2_ in MoS_2_/rGO can be calculated to be 83.24 wt%, which means that the content of rGO is 16.76 wt%.

**Figure 4:**
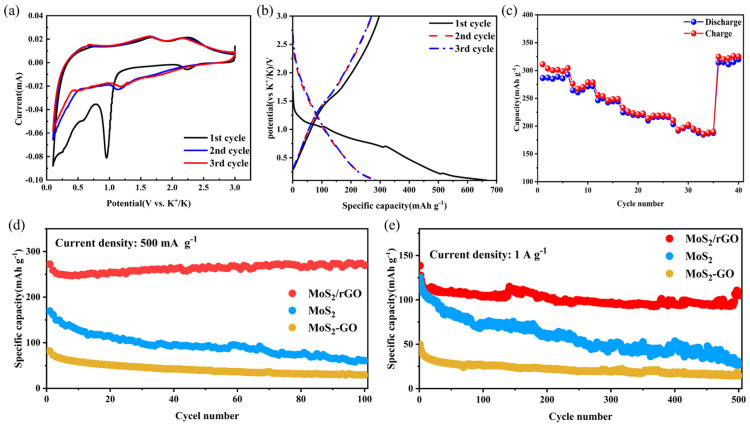
(**a**) Curves of the first three cycles of the MoS_2_/rGO composite electrode at 0.1 mV s^−1^, (**b**) GCD diagram of the first three cycles of the MoS_2_/rGO composite at a current density of 100 mA g^−1^, (**c**) rate performance of the MoS_2_/rGO composite under a different current density of 50, 100, 200, 400, 500, 800, 1000 mA g^−1^, (**d**) 100 long cycle diagram of MoS_2_/rGO composite, MoS_2_, and MoS_2_-GO at a current density of 500 mA g^−1^, (**e**) 500 long cycle diagram of MoS_2_/rGO composite, MoS_2_, and MoS_2_-GO at a current density of 1000 mA g^−1^.

In order to investigate the electrochemical properties of MoS_2_/rGO and MoS_2_ as anode materials, potassium-ion coin cells are fabricated. The electrochemical properties of PIBs consisting of MoS_2_/rGO were determined by cyclic voltammetry (CV) at the working electrode, as shown in Figure 4a. The CV was performed linearly with a sampling frequency of 0.1 mV s^−1^ and a voltage range of 3~0.1 V. The first cathodic scan revealed that one peaks at 0.955 V, indicating the formation and transformation reactions of K_x_MoS_2_ [36]. The anodic scan displayed two peaks at 1.66 V and 2.25 V, which are related to the detachment of K^+^ from the electrode material. As the electrode material interacts with the electrolytic solution during the initial charging and discharging process, a passivation layer is formed on the surface of the electrode, which is referred to as the “solid electrolyte film” or “SEI.” [37,38]. The overlap of the CV curves after the initial cycle indicates the high reversibility of MoS_2_/rGO composite as PIBs during charge/discharge. In contrast, the CV curves of MoS_2_ (Figure S3a) did not exhibit this phenomenon, indicating that MoS_2_ has poor reversibility during the charge–discharge process.

Figure 4b illustrates the constant current charge/discharge (GCD) curve of MoS_2_/rGO at a current density of 100 mA g^−1^. The initial Coulombic efficiency (CE) of MoS_2_/rGO is 44.3%, which is lower than that of MoS_2_ and MoS_2_-GO (Figure S3b,c). This is attributed to the formation of SEI during the initial charge/discharge process, which results in the irreversible reduction of the electrolyte and partially reversible redox reactions of MoS_2_ [39]. The overlapping GCD curves after the initial cycling of MoS_2_/rGO, which was not exhibited by MoS_2_ and MoS_2_-GO, once again demonstrates the high reversibility of MoS_2_/rGO composites for PIBs. As illustrated in Figure S3d, the initial discharge capacity of MoS_2_ is high at 100 mA g^−1^ current density, yet it declines rapidly. After 50 cycles, the specific discharge capacity was only maintained at 171.95 mAh g^−1^. MoS_2_-GO not only has the lowest initial capacity (140.53 mAh g^−1^ at 100 mA g^−1^ current density) but also has a capacity retention of only 40.9% after 50 cycles. MoS_2_/rGO demonstrated the highest initial capacity as well as best capacity retention (discharge capacity was maintained at 349.6 mAh g^−1^ after 50 cycles). The remarkable electrochemical performance is largely attributable to the ultrathin scale structure, enlarged interlayer structure, and excellent intrinsic conductivity of MoS_2_.

The rate performance of MoS_2_/rGO is shown in Figure 4c, showing discharge capacities of 287.15, 266.7, 245.6, 221.6, 215.2, 197.1 and 184.6 mAh g^−1^ at 50, 100, 200, 400, 500, 800, and 1000 mA g^−1^, respectively, which are significantly better than those of MoS_2_ and MoS_2_-GO (Figure S3e,f) When the current density is restored to 50 mA g^−1^, MoS_2_/rGO can recover a specific capacity of 315.02 mAh g^−1^, which is higher than the initial capacity, whereas MoS_2_ and MoS_2_-GO struggle to approach the initial capacity. These results indicate that the unique squamous structure of MoS_2_/rGO can provide capable rate performance.

Figure 4d demonstrates the 100 charge–discharge cycle characteristics of MoS_2_/rGO, MoS_2_, and MoS_2_-GO at 500 mA g^−1^. The initial discharge specific capacity of MoS_2_/rGO is 272.49 mAh g^−1^, while the discharge specific capacity after 100 cycles is 269.5 mAh g^−1^, with a capacity retention of 99%. In contrast, MoS_2_-GO exhibited the lowest initial capacity (81.91 mAh g^−1^) and only 36% capacity retention after 100 cycles, which was slightly higher than that of MoS_2_ (35.91% capacity retention after 100 cycles). The excellent electrochemical performance at high current density is attributed to the scale-like lamellar structure that provides more K-embedding sites, and the enlarged interlayer spacing ensures that the material remains stable during the charging and discharging process [24]. At a high current density of 500 mA g^−1^, the capacity retention of MoS_2_/rGO is close to 99%, indicating that it is more suitable for operation at high current densities. In light of these findings, we sought to ascertain whether MoS_2_/rGO exhibits comparable cycling stability at higher current densities. Figure 4e presents the long weekly cycle plot of MoS_2_/rGO for 500 cycles at a current density of 1 A g^−1^. It can be seen that MoS_2_/rGO is still able to maintain good cycling stability compared to MoS_2_ and MoS_2_-GO, retaining 76% capacity after 500 cycles [40]. In addition, other MoS_2_ anode electrodes are listed in Table 1 and compared to MoS_2_/rGO. Of particular note is the exceptionally high cycling stability of MoS_2_/rGO.

**Figure 5:**
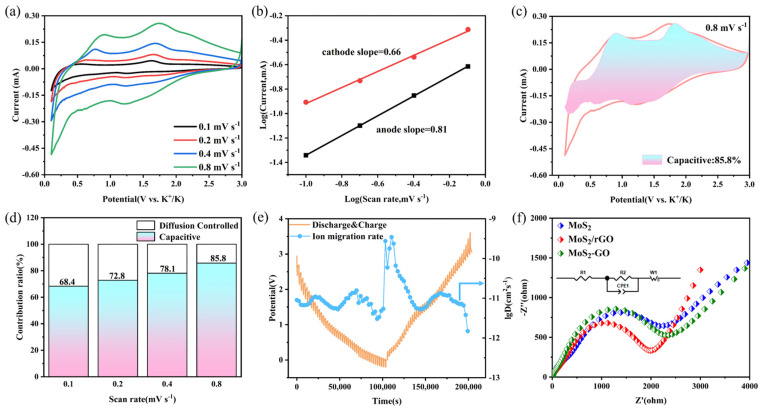
(**a**) CV curves of the MoS_2_/rGO composite at different scan rates from 0.1 to 0.8 mV s^−1^, (**b**) linear fitting of log (peak current) versus log (scan rate) plot of MoS_2_/rGO composite, (**c**) the CV profile with capacitance contribution at 0.8 mV s^−1^, (**d**) the percentages between diffusion and capacitive contribution at different scanning rates for MoS_2_/rGO composite, (**e**) GITT curve of MoS_2_/rGO composite and its calculated ion diffusion coefficient diagram, (**f**) the electrochemical impedance spectrum and equivalent circuit diagram of the MoS_2_/rGO composite, MoS_2_, and MoS_2_-GO.

In order to further study the electrochemical stored procedure of MoS_2_/rGO, CV curves at varying scan rates (ranging from 0.1 to 0.8 mV s^−1^) were tested. It can be observed that MoS_2_/rGO maintains the same shape as the initial redox peaks, whereas MoS_2_ and MoS_2_-GO do not exhibit the same behavior. This suggests that the MoS_2_/rGO exhibit excellent electrochemical reversibility (Figure 5a and Figure S4a,b). Furthermore, the charge storage mechanism was evaluated using power-law analysis [45]:

(1) $$i=a\nu^{b}$$

where ‘*i*’ represents the peak current, ‘*ν*’ represents the scan rate (mV s^−1^), and ‘*a*’ and ‘*b*’ represent constant parameters. A value of ‘*b*’ approaching 1 indicates that capacitive behavior is the dominant phenomenon, whereas a value of ‘*b*’ approaching 0.5 indicates that charge storage is dependent on diffusion processes. As illustrated in Figure 5b, the *b*-values of the anodic and cathodic peaks of MoS_2_/rGO are 0.81/0.66, indicating that the electrochemical potassium storage behavior of the material is primarily governed by the capacitance effect. The *b*-values of the anodic and cathodic peaks of MoS_2_ and MoS_2_-GO are 0.77/0.65 and 0.87/0.69, respectively, and the capacitive contributions are equally dominant (Figure S4c). The contribution of value-diffusive behavior and -capacitive behavior to the capacity can be calculated using the following equation [46]:

(2) $$i=k_{1}v+k_{2}v^{1/2}$$

where *k*_1_, *k*_2_ are constants, and *k*_1_*v* and *k*_2_*v*^1/2^ reflect the contributions of capacitive-controlled and diffusion-controlled processes, respectively. As illustrated in Figure 5c, at a sweep rate of 0.8 mV s^−1^, approximately 85.8% of the total capacitance of MoS_2_/rGO is attributed to the capacitive control process. As the sweep rate increases from 0.1 mV s^−1^ to 0.8 mV s^−1^, the capacitance contribution increases from 68.4% to 85.8% (Figure 5d). These results indicate that the surface diffusion behavior dominates in MoS_2_/rGO. The capacitance contribution of MoS_2_ and MoS_2_-GO is significantly less than that of MoS_2_/rGO (Figure S4d–g). This is primarily due to the unique scale-like lamellar structure of MoS_2_ which provides a greater number of active sites for K^+^ and enhances charge storage. This is also the reason for the excellent rate performance of MoS_2_-GO. The diffusion coefficient of K^+^ during a complete charge/discharge is calculated by the following equation [47]:

(3) $$D=\frac{4}{\pi \tau }{\left(\frac{n_{m}V_{m}}{S}\right)}^{2}{\left(\frac{\Delta E_{s}}{\Delta E_{t}}\right)}^{2}$$

where *τ* is the relaxation time, *n*_m_ is the number of moles, *V*_m_ is the molar volume of the electrode material, *S* is the electrode/electrolyte contact area, the Δ*E*_s_ is the pulse-induced voltage change, and Δ*E*_t_ is the voltage change due to constant current charging (discharging). The calculated results are shown in Figure 5c, and the ion diffusion rate of MoS_2_/rGO is approximately 10^−11^ cm s^−1^, which is comparable to that of other MoS_2_ anodes [28]. Furthermore, the ion diffusion rates of MoS_2_ and MoS_2_-GO were calculated, with values of approximately 10^−12^ cm s^−1^ for MoS_2_ and a range of 10^−12^ to 10^−11^ cm s^−1^ for MoS_2_-GO (Figure S4h,i).

The electrochemical performance of batteries is mainly assessed in terms of the charge transfer capacity and the conductivity of the electrodes [48]. To gain deeper insights into the physical characteristics and outstanding reversible capacity of the MoS_2_/rGO composite electrode, a frequency range of 0.1–100,000 Hz was employed using an electrochemical workstation. Electrochemical impedance spectroscopy (EIS) was performed on a MoS_2_/rGO anode to evaluate its use in PIBs. Figure 5d shows the EIS comparison images of MoS_2_/rGO, MoS_2_, and MoS_2_-GO. The internal resistance of the cell can be analyzed in the high-frequency region, while the mid-frequency region indicates the transfer resistance of the electrode material interface. The larger the ion diffusion coefficient and the steeper the slope, the greater the diffusion capacity. In the equivalent diagram, R1 is the internal resistance, R2 is the interface transfer resistance, CPE1 is the constant phase angle element (similar to a capacitor), and W1 is the Warburg impedance. It can be seen that the semicircular diameter of MoS_2_ and MoS_2_-GO are larger than that of MoS_2_/rGO, and the slope of the diagonal line is also smaller than that of MoS_2_/rGO, which proves that the transfer of K^+^ in MoS_2_ and MoS_2_-GO is slow, while the interfacial transfer resistance of MoS_2_/rGO is relatively small, and the reduction in the charge transfer resistance leads to the increase in the ion diffusion coefficient, and the surface of the electrode is more stabilized [49].

In order to further study the diffusion kinetics of K^+^ in MoS_2_, the de/insertion process of K^+^ was calculated with first principles. All possible embedding positions of K in 2H-MoS_2_, which is an AB-stacked periodic structure, have been scrutinized. K^+^ has been inserted in the AB layer at sites A and B in MoS_2_ (Figure S5a,b). At site A, K^+^ is located in an octahedron of six S atoms. While at site B, K^+^ is located in a tetrahedron of four S atoms. After optimizing the structure, all K^+^ interpolation positions are located at the A site (Figure S5c,d), which indicates that site A is the stable site for K^+^ to insert. The calculation model of MoS_2_/rGO is illustrated in Figure S6a, which comprises three layers of MoS_2_ and three layers of graphene, with a layer spacing of 6.39 Å. The computational model for K^+^ adsorption on the MoS_2_/rGO surface is shown in Figure S5b. Furthermore, the binding and adsorption energies of MoS_2_/rGO were calculated to be −2.46 eV and −3.9 eV, respectively. The high absolute value of the adsorption energy indicates that K^+^ is more favored on the surface of the material, which corresponds to the capacitor behavior described above. Additionally, the negative value suggests that the structure is stabilized, which positively affects the electrochemical properties of the material.

**Figure 6:**
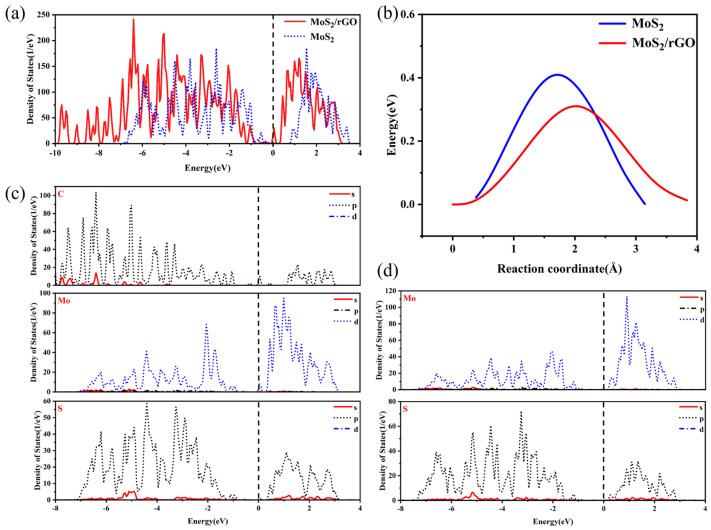
(**a**) TDOS of MoS_2_/rGO composite and MoS_2_, (**b**) diffusion barriers of MoS_2_/rGO composite and MoS_2_, (**c**) PDOS of MoS_2_/rGO composite, (**d**) PDOS of MoS_2_.

Figure 6a illustrates the density of states of MoS_2_ with a band gap of 1.2 eV. Upon the addition of rGO, the band gap of MoS_2_/rGO disappears and crosses the Fermi energy level, indicating that rGO is capable of enhancing the electrical conductivity of the material. It is noteworthy that the projected density of states (PDOS) indicates that the p-orbitals of C prompt the d-orbitals of Mo to cross the Fermi energy level, thereby directly increasing the intrinsic conductivity of MoS_2_ (Figure 6c,d). We considered two migration paths for K in MoS_2_/rGO, one where K^+^ migrates from one octahedral position to neighboring octahedral positions and the other where K^+^ migrates to obliquely oriented octahedral positions (Figure S7). As shown in Figure 6b, the migration potential barrier of K^+^ in MoS_2_/rGO for adjacent octahedral positions is 0.3 eV, which is significantly smaller than that of K^+^ in MoS_2_ (0.4 eV). The results indicate that K^+^ diffuses more readily into neighboring octahedral positions.

## 3. Materials and Methods

### 3.1. Material

Sodium molybdate dihydrate (Na_2_MoO_4_·2H_2_O), CTAB, (C_19_H_42_BrN), ice acetic acid (CH_3_COOH), and anhydrous ethanol (C_2_H_5_OH) were purchased from Sinopharm Chemical Reagent Co., Ltd. (Shanghai, China). Thiourea (CH_4_N_2_S, 99% purity) was purchased from Shanghai Aladdin Industrial Co., Ltd. (Shanghai, China). Single-layer industrial grade graphene oxide was purchased from Suzhou Carbon Feng Technology Co., Ltd. (Suzhou, China).

### 3.2. Preparation of MoS_2_/rGO Composite

An amount of 0.09 g (30 wt%) of GO powder was dissolved in 30 mL of deionized water (DI) and ultrasonicated for 1.5 h (a black-brown solution was generated). Next, 20 mL of N-methyl pyrrolidone was added to the solution and stirred for 10 min. An amount of ice acetic acid was added to the solution, the pH of the solution was adjusted to 6, and the solution was stirred for 5 min to ensure even mixing. CTAB (0.15 g) was added to a 10 mL DI beaker and stirred until it was a milky white solution. Finally, the two solutions were mixed and stirred for 5 h. The two solutions were stratified at the beginning of mixing (the upper layer was graphene oxide, and the lower layer was a transparent liquid), and after 5 h of magnetic stirring, they were uniform solutions with some foam (the nature of CTAB). To the above solution, 0.45 g of Na_2_MoO_4_·2H_2_O and 0.637 g of CH_4_N_2_S were added and continuously stirred for 1 h. Then, the mixture was transferred into a 100 mL high-pressure reactor, kept at 200 °C for 24 h, and allowed to cool to room temperature. The black sediment was collected and rinsed 3 times with DI and anhydrous ethanol. The collection was dried overnight in a vacuum drying oven. Finally, the MoS_2_/rGO composites were obtained by holding the dried black powder at 820 °C for 120 min and at a heating rate of 5 °C min^−1^ under an argon atmosphere.

### 3.3. Structural Characterization

The crystalline phases of the samples were characterized by X-ray diffraction (XRD, XD3, Beijing Puxi Tongyong Co., Ltd., Beijing, China) with Cu-Kα radiation (λ = 1.54 Å). The morphologies and structures of the products were characterized by field-emission scanning electron microscopy (FE-SEM, Quanta250FEG, FEI, OR, USA) and high-resolution transmission electron microscopy (HRTEM, Talos F200, FEI, OR, USA). X-ray absorption fine structure (XAFS) spectra of the powder samples were obtained using 8C. The component and valence analysis of the synthesized samples were carried out by X-ray photoelectron spectroscopy (XPS, ESCALAB Xi+, Thermo Fisher, Morecambe, UK).

### 3.4. Electrochemical Characterization

Active material, acetylene black, and polyvinylidene difluoride (PVDF) at a weight ratio of 8:1:1 was mixed with N-methyl-2 pyrrolidone (NMP) solvent to prepare the anode material. The slurry mass loading on the copper collector was approximately 1 mg cm^−2^. This electrode was dried in a vacuum oven at 50 °C for 12 h. Next, the active electrode was transferred into an Ar-filled glove box to assemble the cell. Coin cells were of the CR2032 type, with a fiberglass to separate the anode and K-block. The electrolyte of choice was 1 M of potassium bis (fluor sulfonyl) imide (KFSI) in ethyl methyl carbonate (EMC). Electrochemical testing of the assembled coin battery was performed at various current densities in the 0.1–3.0 V range by using the Blue Battery Test System. Galvanostatic charge/discharge tests and cyclic voltammetry (CV) measurements in the potential window of 0.1–3.0 V versus K^+^/K and electrochemical impedance spectroscopy (EIS) measurements in the frequency range 0.1 Hz to 100 kHz at open-circuit voltage were performed using an electrochemical workstation (CS350 Coster Co., Ltd., Wuhan, China). All electrochemical measurements were performed at room temperature.

### 3.5. Theoretical Calculation

The calculations in this work are based on the DS-PAW, as well as the Projected Augmented Wave (PAW) method for calculation [50,51]. Ultra-soft pseudopotentials described the interaction between ionic nuclei and valence electrons. This study considers the binding energy, density of states, and migration barrier of MoS_2_/rGO. The exchange and correlation terms were computed using the generalized gradient approximation (GGA) of the Perdew–Burke–Ernzerh method, which was parameterized by Perdew [52,53]. Brillouin zone integrations were performed using a Gamma-centered k-point grid. MoS_2_ exhibits hexagonal symmetry and belongs to the P63/MMC space group.

The calculation was performed using a cutoff energy of 480 eV and a 3 × 3 × 1 k-point grid to ensure total energy convergence. The calculations were considered converged when the maximum force on the atoms was below 0.05 eVÅ^−1^, the maximum stress was below 0.1 GPa, and the maximum displacement between cycles was below 0.002 Å. Periodic structures are utilized in the calculations. To calculate the binding energy, density of states, and migration barriers, we extended MoS_2_ to 3 × 3 × 3 and graphene to 4 × 4 × 4, and used this as the basis for constructing the MoS_2_/rGO model of MoS_2_-wrapped rGO. The binding energy is calculated using the following formula:

(4) $$E_{binding}=E_{MoS_{2}/rGO}-E_{MoS_{2}}-E_{rGO}$$

The adsorption energy is calculated as follows:

(5) $$E_{adsorption}=E_{MoS_{2}/rGO}-E_{MoS_{2}}-E_{rGO}-E_{K}$$

The calculation of the migration barriers took into account the distinct migration paths of K^+^ in MoS_2_/rGO and MoS_2_.

## 4. Conclusions

In summary, MoS_2_/rGO composites were successfully prepared via a one-step hydrothermal method. Owing to the presence of rGO in the synthesis process, the MoS_2_ anodes in such composite possess a unique scaled structure with larger layer spacing, compared with pure MoS_2_. This indirectly increases the specific surface area, provides more active sites for K^+^, and ensures structural stability. In addition, after the hydrothermal reaction, the incorporation of rGO can increase the intrinsic conductivity of MoS_2_, and the composite shows high electronic conductivity.

As a result, MoS_2_/rGO composite anodes exhibited a better rate performance (287.15, 266.7, 220.3, and 184.6 mAh g^−1^, at 50, 100, 500, and 1000 mA g^−1^, respectively) and cycle stability (99% capacity retention after 100 cycles at 500 mA g^−1^) than that of anodes based on pure MoS_2_ and MoS_2_-GO. This work demonstrates the significant potential of MoS_2_/rGO in high-current PIBs and presents a practical strategy for the development of high-performance anode materials for PIBs.

## Figures and Tables

**Table 1:** The comparison with previous work on the MoS_2_-based electrodes for PIBs.

Chemical Formula	Electrochemical Performance	Cycle Number
MoS_2_ [41]	102 mAh g^−1^ (100 mAg^1^)	86 mAh g^−1^ (200 cycles)
MoS_2_@C [42]	~290 mAh g^−1^ (500 mAg^1^)	241 mAh g^−1^ (100 cycles)
MoS_2_@Cnanosheets [43]	300 mAh g^−1^ (100 mA g^−1^)	164.5 mAh g^−1^ (350 cycles)
MoS_2_/MXene [44]	271.4 mAh g^−1^ (50 mA g^−1^)	206 mAh g^−1^ (100 cycles)
C-MoS_2_ [39]	~340 mAh g^−1^ (1000 mA g^−1^)	273 mAh g^−1^ (100 cycles)
This work	272.49 mAh g^−1^ (500 mA g^−1^)	269.5 mAh g^−1^ (100 cycles)

## Data Availability

The data presented in this study are available in the Supplementary Materials.

## Supplementary Materials

The following supporting information can be downloaded at: https://www.mdpi.com/article/10.3390/molecules29132977/s1, Figure S1: X-ray photoelectron spectroscopy (XPS) of MoS_2_ (a) C1s, (b) Mo 3d and (c) S 2p, XPS of MoS_2_-GO (d) C 1s, (e) Mo 3d and (f) S 2p; Figure S2: The thermogravimetric analysis for MoS_2_/rGO; Figure S3: (a) Curves of the first three cycles of the MoS_2_ electrode at 0.1 mV s^−1^, Specific capacity voltage diagram of the first three cycles of (b) MoS_2_ and (c) MoS_2_-GO at a current density of 100 mA g^−1^, (d) 50 long cycle diagrams of MoS_2_/rGO, MoS_2_ and MoS_2_-GO cycle performance at 100 mA g^−1^, rate performance of (e) the MoS_2_ and (f) the MoS_2_-GO under a different current density of 50, 100, 200, 400, 500, 800, 1000 mA g^−1^; Figure S4: (a) CV curves of the MoS_2_ at different scan rates from 0.1 to 0.8 mV s^−1^, (b) CV curves of the MoS_2_-GO at different scan rates from 0.1 to 0.8 mV s^−1^, (c) linear fitting of log (peak current) versus log (scan rate) plot of MoS_2_ and MoS_2_-GO, (d) percentage capacitive contributions of MoS_2_ at 0.8 mV s^−1^, (e) percentage capacitive contributions of MoS_2_-GO at 0.8 mV s^−1^, (f) percentage capacitive contributions of MoS_2_ at different scan rates, (g) percentage capacitive contributions of MoS_2_-GO at different scan rates, (h) GITT curve of MoS_2_ and its calculated ion diffusion coefficient diagram, (i) GITT curve of MoS_2_-GO and its calculated ion diffusion coefficient diagram; Figure S5: (a,b) Possible insertion sites for K before structure optimization, (c,d) sites of K insertion after structural optimization; Figure S6: (a) Calculation model of MoS_2_/rGO, (b) adsorption calculation model of MoS_2_/rGO; Figure S7: Two diffusion paths of MoS_2_/rGO.

## References

[1] Sui Y., Zhou J., Wang X., Wu L., Zhong S., Li Y. (2021). Recent Advances in Black-Phosphorus-Based Materials for Electrochemical Energy Storage. Mater. Today.

[2] Zhou G., Xu L., Hu G., Mai L., Cui Y. (2019). Nanowires for Electrochemical Energy Storage. Chem. Rev..

[3] De Rosa M., Afanaseva O., Fedyukhin A.V., Bianco V. (2021). Prospects and Characteristics of Thermal and Electrochemical Energy Storage Systems. J. Energy Storage.

[4] Wang C.-Y., Liu T., Yang X.-G., Ge S., Stanley N.V., Rountree E.S., Leng Y., McCarthy B.D. (2022). Fast Charging of Energy-Dense Lithium-Ion Batteries. Nature.

[5] Abu S.M., Hannan M.A., Hossain Lipu M.S., Mannan M., Ker P.J., Hossain M.J., Mahlia T.M.I. (2023). State of the Art of Lithium-Ion Battery Material Potentials: An Analytical Evaluations, Issues and Future Research Directions. J. Clean. Prod..

[6] Chayambuka K., Mulder G., Danilov D.L., Notten P.H.L. (2018). Sodium-Ion Battery Materials and Electrochemical Properties Reviewed. Adv. Energy Mater..

[7] Guo Q., Zeng W., Liu S.-L., Li Y.-Q., Xu J.-Y., Wang J.-X., Wang Y. (2021). Recent Developments on Anode Materials for Magnesium-Ion Batteries: A Review. Rare Met..

[8] Das S.K., Mahapatra S., Lahan H. (2017). Aluminium-Ion Batteries: Developments and Challenges. J. Mater. Chem. A.

[9] Min X., Xiao J., Fang M., Wang W., Zhao Y., Liu Y., Abdelkader A.M., Xi K., Kumar R.V., Huang Z. (2021). Potassium-Ion Batteries: Outlook on Present and Future Technologies. Energy Environ. Sci..

[10] Zhang W., Yin J., Wang W., Bayhan Z., Alshareef H.N. (2021). Status of Rechargeable Potassium Batteries. Nano Energy.

[11] Hwang J.-Y., Myung S.-T., Sun Y.-K. (2018). Recent Progress in Rechargeable Potassium Batteries. Adv. Funct. Mater..

[12] Eftekhari A., Jian Z., Ji X. (2017). Potassium Secondary Batteries. ACS Appl. Mater. Interfaces.

[13] Rajagopalan R., Tang Y., Ji X., Jia C., Wang H. (2020). Advancements and Challenges in Potassium Ion Batteries: A Comprehensive Review. Adv. Funct. Mater..

[14] Zhang J., Liu T., Cheng X., Xia M., Zheng R., Peng N., Yu H., Shui M., Shu J. (2019). Development Status and Future Prospect of Non-Aqueous Potassium Ion Batteries for Large Scale Energy Storage. Nano Energy.

[15] Liu S., Kang L., Henzie J., Zhang J., Ha J., Amin M.A., Hossain M.S.A., Jun S.C., Yamauchi Y. (2021). Recent Advances and Perspectives of Battery-Type Anode Materials for Potassium Ion Storage. ACS Nano.

[16] Lei Y., Chen M., Li Y., Zhang W., Zhao D., Zhu Q. (2023). Dendrite-Free Potassium Metal Anode Induced by in-Situ Phase Transitions of MoS_2_. Mater. Today Phys..

[17] Sha M., Liu L., Zhao H., Lei Y. (2020). Anode Materials for Potassium-Ion Batteries: Current Status and Prospects. Carbon Energy.

[18] Presolski S., Pumera M. (2016). Covalent Functionalization of MoS_2_. Mater. Today.

[19] Wang Y.-Z., Shan X.-Y., Wang D.-W., Sun Z.-H., Cheng H.-M., Li F. (2018). A Rechargeable Quasi-Symmetrical MoS_2_ Battery. Joule.

[20] Li C., Lao B., Li Z., Yin H., Yang Z., Wang H., Chen D., Zhang X., Xu Y., Sun C. (2020). Dual-Ion Battery with MoS_2_ Cathode. Energy Storage Mater..

[21] Wu K., Cao X., Li M., Lei B., Zhan J., Wu M. (2020). Bottom-Up Synthesis of MoS_2_/CNTs Hollow Polyhedron with 1T/2H Hybrid Phase for Superior Potassium-Ion Storage. Small.

[22] Yao K., Xu Z., Ma M., Li J., Lu F., Huang J. (2020). Densified Metallic MoS_2_/Graphene Enabling Fast Potassium-Ion Storage with Superior Gravimetric and Volumetric Capacities. Adv. Funct. Mater..

[23] Cui Y., Liu W., Feng W., Zhang Y., Du Y., Liu S., Wang H., Chen M., Zhou J. (2020). Controlled Design of Well-Dispersed Ultrathin MoS 2 Nanosheets inside Hollow Carbon Skeleton: Toward Fast Potassium Storage by Constructing Spacious “Houses” for K Ions. Adv. Funct. Mater..

[24] Liu Y., Xiao Y., Liu F., Han P., Qin G. (2019). Controlled Building of Mesoporous MoS_2_ @MoO_2_-Doped Magnetic Carbon Sheets for Superior Potassium Ion Storage. J. Mater. Chem. A.

[25] Krishnamoorthy K., Veerapandian M., Yun K., Kim S.-J. (2013). The Chemical and Structural Analysis of Graphene Oxide with Different Degrees of Oxidation. Carbon.

[26] Xie K., Yuan K., Li X., Lu W., Shen C., Liang C., Vajtai R., Ajayan P., Wei B. (2017). Superior Potassium Ion Storage via Vertical MoS_2_ “Nano-Rose” with Expanded Interlayers on Graphene. Small.

[27] Pei B., Jiang Z., Zhang W., Yang Z., Manthiram A. (2013). Nanostructured Li3V2(PO4)3 Cathode Supported on Reduced Graphene Oxide for Lithium-Ion Batteries. J. Power Sources.

[28] Ma G., Zhou Y., Wang Y., Feng Z., Yang J. (2021). N, P-Codoped Graphene Supported Few-Layered MoS_2_ as a Long-Life and High-Rate Anode Materials for Potassium-Ion Storage. Nano Res..

[29] Wang J., Fang W., Hu Y., Zhang Y., Dang J., Wu Y., Chen B., Zhao H., Li Z. (2021). Single Atom Ru Doping 2H-MoS_2_ as Highly Efficient Hydrogen Evolution Reaction Electrocatalyst in a Wide pH Range. Appl. Catal. B Environ..

[30] Wurst K.M., Strolka O., Hiller J., Keck J., Meixner A.J., Lauth J., Scheele M. (2023). Electronic Structure of Colloidal 2H-MoS_2_ Mono and Bilayers Determined by Spectroelectrochemistry. Small.

[31] Huang H.-H., De Silva K.K.H., Kumara G.R.A., Yoshimura M. (2018). Structural Evolution of Hydrothermally Derived Reduced Graphene Oxide. Sci. Rep..

[32] Liu M., Liu Y., Tang B., Zhang P., Yan Y., Liu T. (2017). 3D Conductive Network Supported Monolithic Molybdenum Disulfide Nanosheets for High-Performance Lithium Storage Applications. Adv. Mater. Inter..

[33] Hu X., Li Y., Zeng G., Jia J., Zhan H., Wen Z. (2018). Three-Dimensional Network Architecture with Hybrid Nanocarbon Composites Supporting Few-Layer MoS_2_ for Lithium and Sodium Storage. ACS Nano.

[34] Gao J., Li Y., Liu Y., Jiao S., Li J., Wang G., Zeng S., Zhang G. (2019). The Dual-Function Sacrificing Template Directed Formation of MoS_2_/C Hybrid Nanotubes Enabling Highly Stable and Ultrafast Sodium Storage. J. Mater. Chem. A.

[35] Xu Z., He M., Zhou Y., Nie S., Wang Y., Huo Y., Kang Y., Wang R., Xu R., Peng H. (2021). Spider Web-like Carbonized Bacterial Cellulose/MoSe2 Nanocomposite with Enhanced Microwave Attenuation Performance and Tunable Absorption Bands. Nano Res..

[36] Jia B., Yu Q., Zhao Y., Qin M., Wang W., Liu Z., Lao C.-Y., Liu Y., Wu H., Zhang Z. (2018). Bamboo-Like Hollow Tubes with MoS_2_/N-Doped-C Interfaces Boost Potassium-Ion Storage. Adv. Funct. Mater..

[37] Wang H., Zhai D., Kang F. (2020). Solid Electrolyte Interphase (SEI) in Potassium Ion Batteries. Energy Environ. Sci..

[38] Gu M., Rao A.M., Zhou J., Lu B. (2023). In Situ Formed Uniform and Elastic SEI for High-Performance Batteries. Energy Environ. Sci..

[39] Zhang Y., Zhu L., Xu H., Wu Q., Duan H., Chen B., He H. (2023). Interlayer-Expanded MoS_2_ Enabled by Sandwiched Monolayer Carbon for High Performance Potassium Storage. Molecules.

[40] Liu H., He Y., Cao K., Wang S., Jiang Y., Liu X., Huang K.-J., Jing Q.-S., Jiao L. (2021). Stimulating the Reversibility of Sb2S3 Anode for High-Performance Potassium-Ion Batteries. Small.

[41] Fagiolari L., Versaci D., Di Berardino F., Amici J., Francia C., Bodoardo S., Bella F. (2022). An Exploratory Study of MoS_2_ as Anode Material for Potassium Batteries. Batteries.

[42] Chen L., Chen Z., Chen L., Zhou P., Wang J., Yang H., Feng Z., Li X., Huang J. (2023). The Exfoliation of Bulk MoS_2_ by a Three-Roller Mill for High-Performance Potassium Ion Batteries. Appl. Surf. Sci..

[43] Zhang J., Cui P., Gu Y., Wu D., Tao S., Qian B., Chu W., Song L. (2019). Encapsulating Carbon-Coated MoS_2_ Nanosheets within a Nitrogen-Doped Graphene Network for High-Performance Potassium-Ion Storage. Adv. Mater. Interfaces.

[44] Li J., Rui B., Wei W., Nie P., Chang L., Le Z., Liu M., Wang H., Wang L., Zhang X. (2020). Nanosheets Assembled Layered MoS_2_/MXene as High Performance Anode Materials for Potassium Ion Batteries. J. Power Sources.

[45] Kim H., Byeon Y.-W., Wang J., Zhang Y., Scott M.C., Jun K., Cai Z., Sun Y. (2022). Understanding of Electrochemical K+/Na+ Exchange Mechanisms in Layered Oxides. Energy Storage Mater..

[46] Ma M., Zhang S., Yao Y., Wang H., Huang H., Xu R., Wang J., Zhou X., Yang W., Peng Z. (2020). Heterostructures of 2D Molybdenum Dichalcogenide on 2D Nitrogen-Doped Carbon: Superior Potassium-Ion Storage and Insight into Potassium Storage Mechanism. Adv. Mater..

[47] Fang G., Wu Z., Zhou J., Zhu C., Cao X., Lin T., Chen Y., Wang C., Pan A., Liang S. (2018). Observation of Pseudocapacitive Effect and Fast Ion Diffusion in Bimetallic Sulfides as an Advanced Sodium-Ion Battery Anode. Adv. Energy Mater..

[48] Zhao J., Burke A.F. (2021). Electrochemical Capacitors: Materials, Technologies and Performance. Energy Storage Mater..

[49] Wang S., Zhang J., Gharbi O., Vivier V., Gao M., Orazem M.E. (2021). Electrochemical Impedance Spectroscopy. Nat. Rev. Methods Primers.

[50] Blöchl P.E. (1994). Projector Augmented-Wave Method. Phys. Rev. B.

[51] Kresse G., Joubert D. (1999). From Ultrasoft Pseudopotentials to the Projector Augmented-Wave Method. Phys. Rev. B.

[52] Marlo M., Milman V. (2000). Density-Functional Study of Bulk and Surface Properties of Titanium Nitride Using Different Exchange-Correlation Functionals. Phys. Rev. B.

[53] White J.A., Bird D.M. (1994). Implementation of Gradient-Corrected Exchange-Correlation Potentials in Car-Parrinello Total-Energy Calculations. Phys. Rev. B.

