# Supplementary material for: Scaly MoS2/rGO Composite as an Anode Material for High-Performance Potassium-Ion Battery
Source: Molecules. 2024 Jun 22;29(13):2977. doi: 10.3390/molecules29132977 (PMC11243079; doi:10.3390/molecules29132977)
Supplement: Supplementary file 1 [file molecules-29-02977-s001.zip › molecules-3048315-supplementary.pdf]

Supporting Information

Scaly MoS_2/rGO Composite as an Anode Material for High-Performance Potassium-Ion Battery

Bin Wang [1,2,3], Tao Deng [1,2], Jingjing Liu [1], Beibei Sun [1,3], Yun Su [1,3], Ruixia Ti [1,3], Lihua Shangguan [1], Chaoyang Zhang [1], Yu Tang [1,2], Na Cheng [1], Yan Xu [1,2,3,*] and Junling Guo [4,*]

[1] School of Physics and Electronic Engineering, Xinxiang University, Xinxiang 453003, China; wangbin2013@xxu.edu.cn (B.W.); dengtaoxuyiyin@163.com (T.D.); bit-1@163.com (Y.S.); tiruixia@yeah.net (R.T.)
[2] School of Mechanical Engineering, Chengdu University, Chengdu 610106, China
[3] Henan Province Engineering Research Center of New Energy Storage System, Xinxiang University, Xinxiang 453003, China
[4] Country State Center for International Cooperation on Designer Low-Carbon & Environmental Materials, School of Materials Science and Engineering, Zhengzhou University, 100 Kexue Avenue, Zhengzhou 450001, China
[*] Correspondence: xuyanchenchengshi@126.com (Y.X.); guojunling@zzu.edu.cn (J.G.)

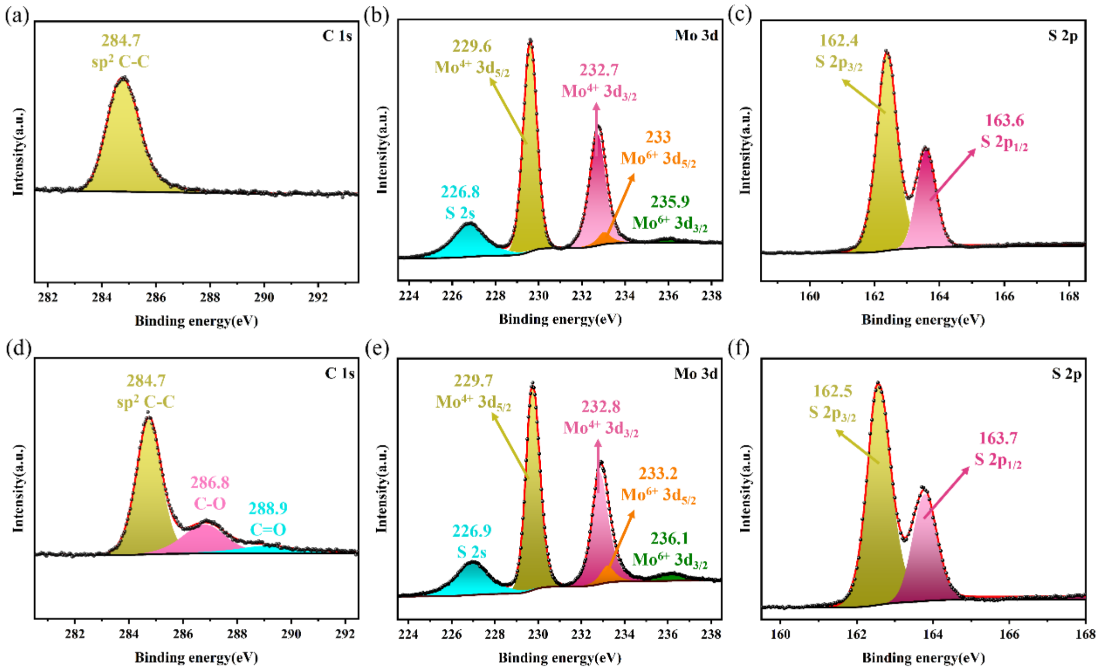

Figure S1. X-ray photoelectron spectroscopy (XPS) of MoS_2 **(a)** C1s, **(b)** Mo 3d and **(c)** S 2p. XPS of MoS_2-GO **(d)** C 1s, **(e)** Mo3d and **(f)** S 2p.

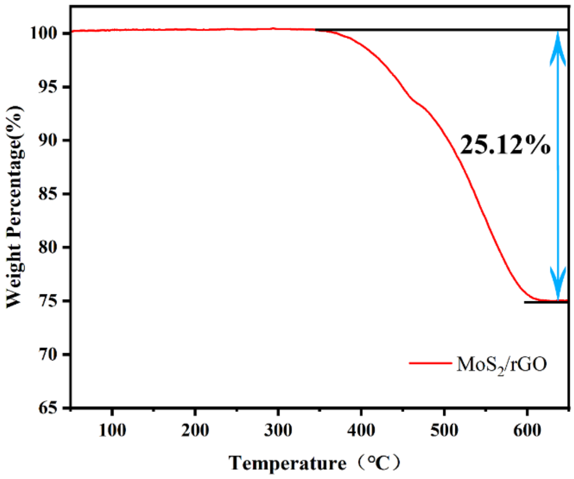

Figure S2. The thermogravimetric analysis for MoS_2/rGO.

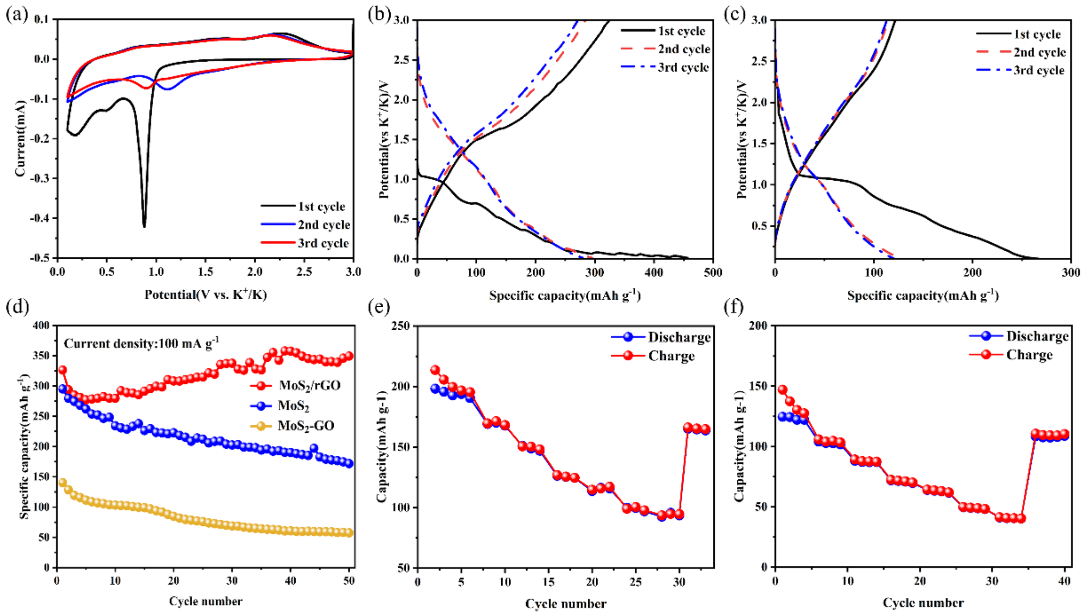

Figure S3. (a) Curves of the first three cycles of the MoS_2 electrode at 0.1 mV s^{-1}, Specific capacity voltage diagram of the first three cycles of **(b)** MoS_2 and **(c)** MoS_2-GO at a current density of 100 mA g^{-1}, **(d)** 50 long cycle diagrams of MoS_2/rGO, MoS_2 and MoS_2-GO cycle performance at 100 mA g^{-1}, rate performance of **(e)** the MoS_2 and **(f)** the MoS_2-GO under a different current density of 50, 100, 200, 400, 500, 800, 1000 mA g^{-1}.

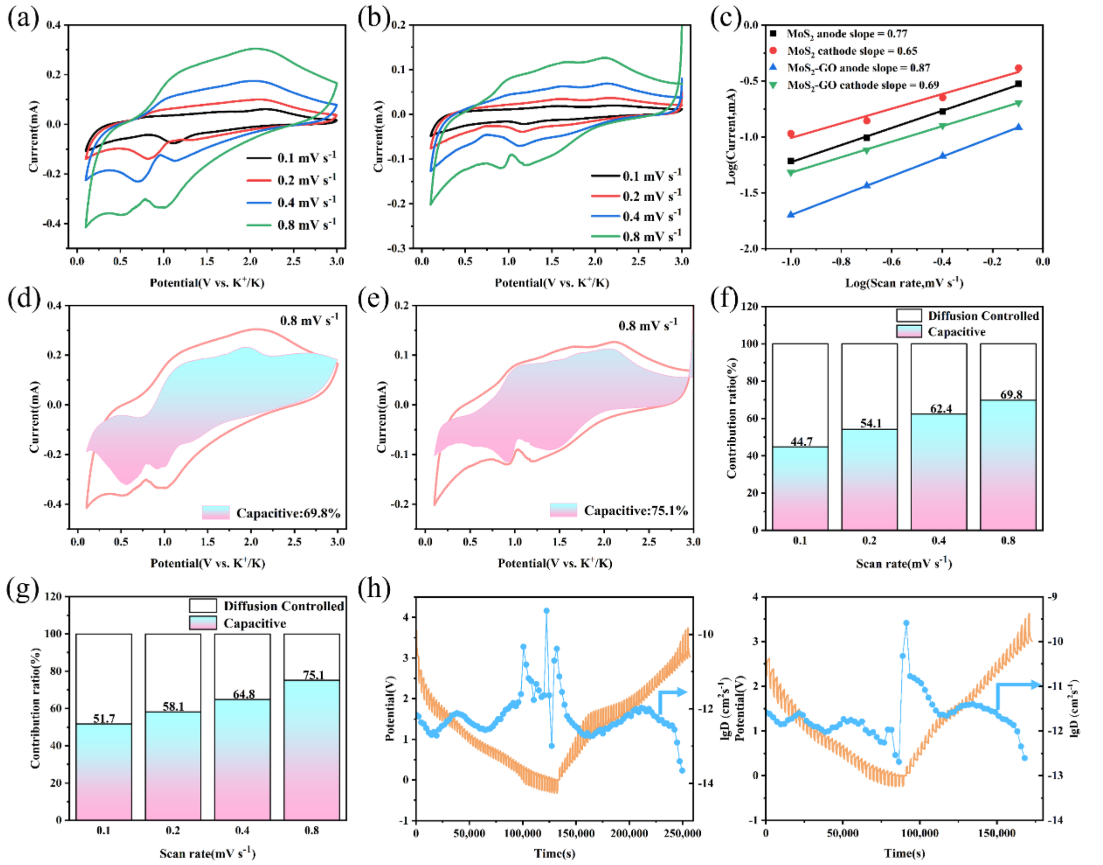

Figure S4. **(a)** CV curves of the MoS_2 at different scan rates from 0.1 to 0.8 mV s^{-1}, **(b)** CV curves of the MoS2-GO at different scan rates from 0.1 to 0.8 mV s^{-1}, **(c)** linear fitting of log (peak current) versus log (scan rate) plot of MoS_2 and MoS_2-GO, **(d)** percentage capacitive contributions of MoS_2 at 0.8 mV s^{-1}, **(e)** percentage capacitive contributions of MoS_2-GO at 0.8 mV s^{-1}, **(f)** percentage capacitive contributions of MoS_2 at different scan rates, **(g)** percentage capacitive contributions of MoS_2-GO at different scan rates, **(h)** GITT curve of MoS_2 and its calculated ion diffusion coefficient diagram, **(i)** GITT curve of MoS_2-GO and its calculated ion diffusion coefficient diagram.

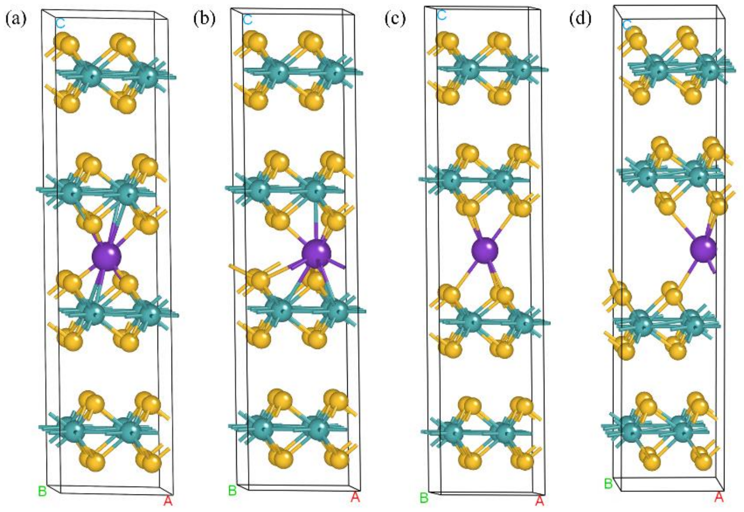

Figure S5. **(a, b)** Possible insertion sites for K before structure optimization, **(c, d)** sites of K insertion after structural optimization.

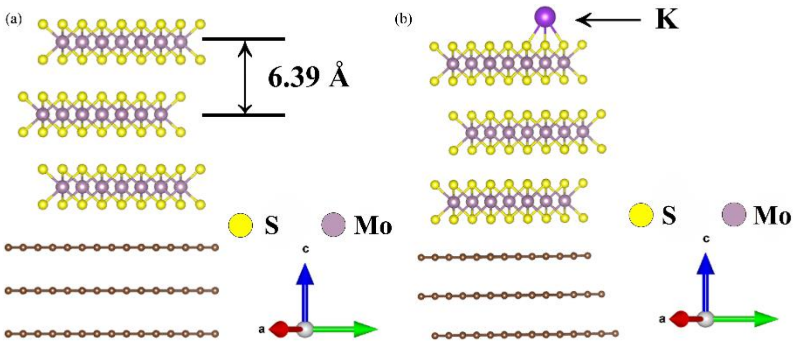

Figure S6. (a) Calculation model of MoS_2/rGO, **(b)** adsorption calculation model of MoS_2/rGO.

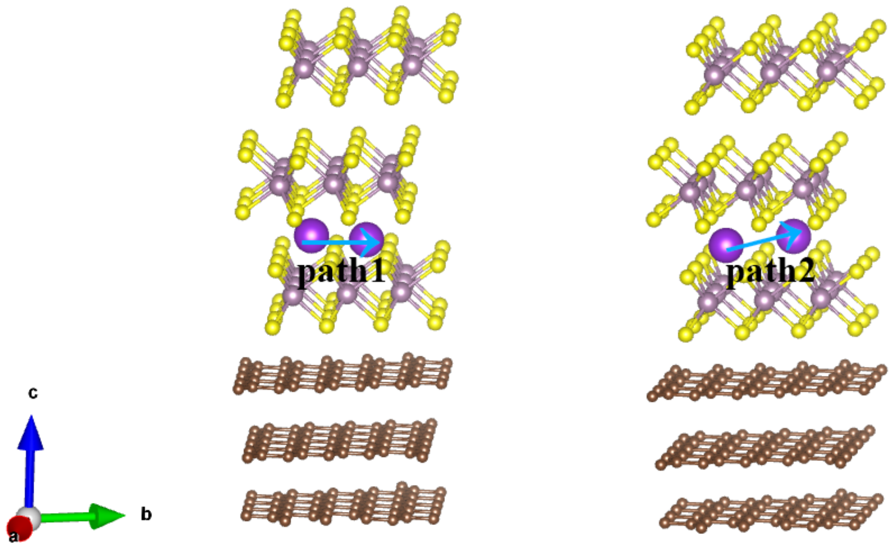

Figure S7. Two diffusion paths of MoS_2/rGO.
